# Serum Gamma-Glutamyltransferase Levels are Associated with Cardiovascular Risk Factors in China: A Nationwide Population-Based Study

**DOI:** 10.1038/s41598-018-34913-7

**Published:** 2018-11-08

**Authors:** Dan-Dan Li, Tao Xu, Xin-Qi Cheng, Wei Wu, Yi-Cong Ye, Xiu-Zhi Guo, Qian Cheng, Qian Liu, Li Liu, Guang-Jin Zhu, Jie Wu, Ling Qiu

**Affiliations:** 1 Department of Clinical Laboratory, Peking Union Medical College Hospital, Peking Union Medical College & Chinese Academy of Medical Science, Beijing, 100730 China; 2 Department of Statistics, Institute of Basic Medical Sciences, Chinese Academy of Medical Sciences & Peking Union Medical College, Beijing, 100005 China; 3 Department of Cardiology, Peking Union Medical College Hospital, Peking Union Medical College & Chinese Academy of Medical Sciences, Beijing, 100730 China; 4 Department of Pathophysiology, Institute of Basic Medical Sciences, Chinese Academy of Medical Sciences & Peking Union Medical College, Beijing, 100005 China

## Abstract

Serum gamma-glutamyltransferase (GGT), which is mainly derived from the liver, is a sensitive marker of liver cell damage and oxidative stress. More recently, it has been found that increased GGT plasma activity is also associated with cardiovascular disease (CVD). However, data on the relationship between GGT and cardiovascular risk factors (CRFs) are lacking in nationally representative samples of the Chinese population. Here, we aim to investigate both the association between GGT and CRFs and CRF clustering. A cross-sectional survey was conducted in a representative sample of 22897 adults aged 18 years and older from 2007 to 2011 nationally, which included a plurality of ethnic minorities. The participants were then divided into quartiles of sex-specific serum GGT. From the low to high GGT quartiles, the incidence of each CRF and clustered risk factors increased after adjusting for age, uric acid (UA), ethnicity, drinking, and all other risk factors. Individuals in the upper stratum (>75^th^ percentile) had higher prevalence rates of CRFs than did those in the lower stratum (all P < 0.05). Furthermore, the subjects with clustering of 1, 2, or ≥3 CRFs were still more likely to belong to the upper GGT quartiles (75th percentiles) than were those without risk factors (all P < 0.05). In conclusion, our data highlight that there is an association between higher serum GGT levels and prevalence of CRFs, which tend to cluster with the increase in GGT activity in Chinese adults.

## Introduction

Cardiovascular disease (CVD) is considered a major cause of death in most developed and developing countries^1^ and represents a huge economic burden for humans^2^. Our previous study showed that the general prevalence estimates of cardiovascular risk factors (CRFs), including diabetes, dyslipidaemia, hypertension, overweight/obesity, and smoking, are high in China, covering all adult age groups by race/ethnicity^3^. It is recognized that the more risk factors there are in the same individual, the more the incidence of future CVD will increase compared with individuals with only a single risk factor^4^.

Gamma-glutamyltransferase (GGT) is a liver enzyme that is traditionally used in clinical practice as a marker for liver function and alcohol abuse^5^. However, the predictive utility of GGT applies well beyond liver disease. Serum GGT is not only a correlate of increased risk for cardiovascular death, but is also associated with higher risk of premature coronary artery disease (CAD) in young patients with typical chest pain or positive non-invasive tests^6^. Several longitudinal and cross-sectional investigations have associated GGT with an increase in all-cause mortality and chronic heart diseases such as congestive heart failure^7,8^. Recent studies have also shown that elevated serum GGT levels are a risk factor for cardiovascular deaths in both healthy and type 2 diabetic patients^9^. In addition, elevated serum levels of GGT are associated with metabolic syndrome^10^, and metabolic syndrome itself can promote the development of atherosclerosis and CVD. Baseline circulating GGT levels and an increase in GGT over time are associated with an increased risk of hypertension^11,12^. This association is stronger in obese men and women than in their lean counterparts^13^.

Therefore, a number of studies have confirmed that elevated GGT levels are linked to an increased risk of a multitude of diseases and conditions, including CVD and CRFs, such as diabetes, metabolic syndrome (MetS), hypertension, and all-cause mortality^14,15^.

Previous studies on the Chinese population focused more on the correlation between GGT and a single CRF, and such studies were limited to certain geographical regions, so the population was not representative^16,17^. China, as a large developing country, has marked regional and ethnic differences. In addition, the levels of GGT differ between districts and counties. Moreover, there is also little literature reporting the relationship between serum GGT and CRF clustering in the Chinese population. Therefore, a nationally representative study about the associations between serum GGT and CRFs, as well as CRF clustering in China, was conducted.

## Methods

### Study Design and Participants

**Figure 1:**
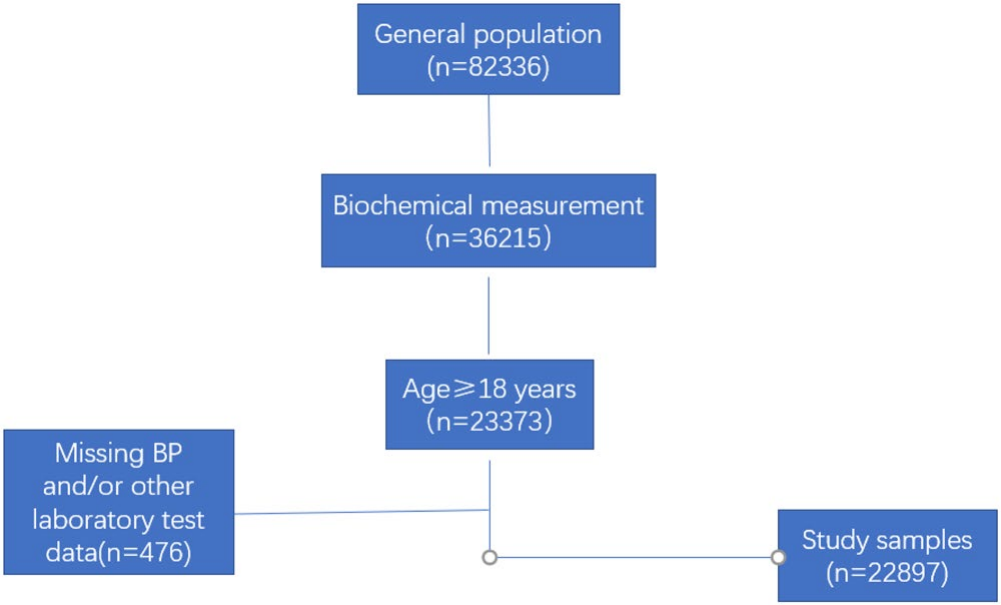
A schematic used for screening and inclusion of study samples. The final sample size was 22897, which consisted of 10715 men and 12182 women. BP = blood pressure.

We conducted a population-based, cross-sectional survey from 2007 to 2011. The data used in this study are from the Chinese Physiological Constant and Health Condition (CPCHC) survey. This research used a multistage, random, stratified sampling method to obtain a nationally representative sample of the general Chinese population^18,19^. In brief, 6 provinces (Sichuan-Southwest, Yunnan-South, Hunan-South, Inner Mongolia Autonomous Region-North, Ningxia Hui Autonomous Region-Northwest, and Heilongjiang-Northeast) were selected from different geographical regions in China. A 3-stage cluster sampling method was used to select eligible subjects in each province. 82336 apparently healthy participants aged 10–80 years old who were not suffering from severe systemic diseases (such as cancer, pulmonary disease, renal, cardiovascular or gastrointestinal) or did not have a high fever in the past two weeks were included. Written informed consent forms were obtained from each participant prior to data collection, and participants reported to the physical examination centers voluntarily to take part in the survey. Figure 1 shows the schematic of the screening process. There were 82336 eligible subjects, among which 36215 people were selected randomly to complete blood biochemical testing. Finally, 23373 subjects (64.5%) were included after excluding subjects aged <18 years. Of the 23373, 476 (2.0%) adults had missing blood pressure (BP), GGT, and/or other blood sample parameters. Actually, the final sample size in this study included 22897 people (10715 men and 12182 women). The protocol was in accordance with the Helsinki Declaration and was approved by the institutional review board of the Institute of Basic Medical Sciences, Chinese Academy of Medical Sciences.

### Questionnaire and Physical Examination

All of the participants were asked to complete a standard questionnaire. Measure body weight to the nearest 0.1 kg on a calibrated beam scale, and measure height to the nearest 0.1 cm barefoot in triplicate using a wall-mounted stadiometer. Body mass index (BMI, an index for overall obesity) was calculated as body weight divided by height (in square meters). Waist circumference (WC) was measured midway between the lower rib margin and the iliac crest. Blood pressure was measured after the participant had rested quietly for at least 10 minutes, using an electric sphygmomanometer (OMRON, HEM-7000). We took the measurements three times and used the averages. All personnel who took part in data collection and anthropometry were trained medical personnel, and all devices were calibrated.

### Laboratory Measurements

All procedures were performed following a 9–12 hour overnight fast. The blood specimens were centrifuged, and the serum was stored at -80 °C until the measurements were performed. High-density lipoprotein cholesterol (HDL-C), triglycerides (TG), total cholesterol (TC), and low-density lipoprotein cholesterol (LDLC) were tested with a Beckman AU Series Automatic Biochemical Analyzer (Japan), and the Sekisui Medical (Japan) reagents were used.

GGT, fasting blood glucose (FBG), creatine, uric acid (UA), and other biochemical tests were measured with the same instrument. The Peking Union Medical College Hospital took charge of the common internal quality control program which was followed by the biochemical laboratories participating in the survey. Estimated Glomerular Filtration Rate (eGFR) was calculated by the CG/BSA formula^20^.

### Study definitions

Hypertension was defined by the Seventh Report of the Joint National Committee on Prevention, Detection, Evaluation, and Treatment of High Blood Pressure (JNC7)^21^ as having an average systolic blood pressure (SBP) ≥ 140 mm Hg and/or diastolic blood pressure (DBP) ≥ 90 mm Hg. It was also defined by the 2017 ACC/AHA as having an average SBP ≥130 mm Hg and/or DBP ≥80 mm Hg, and/or current anti-hypertensive medication use^22^. Diabetes was diagnosed as FBG ≥7.0 mmol/L and/or current anti-diabetes medication use. Dyslipidemia was defined as having at least one of the following: TC ≥5.2 mmol/L, TG ≥1.7 mmol/L, HDL-C < 1.0 mmol/L, LDL-C ≥3.4 mmol/L, and/or current cholesterol-lowering medication use. Being overweight and obese were defined as having a BMI ≥25 kg/m^2^ and BMI ≥30 kg/m^2^, respectively. CRF clustering was categorized as 0 risk factor, ≥1 risk factors, ≥2 risk factors, and ≥3 risk factors.

In this study, we evaluated five risk factors including hypertension, diabetes, dyslipidemia, overweight, smoking in the study population and analyzed the relationship between serum GGT and these risk factors in the Chinese population.

### Statistical Analysis

Normally distributed continuous variables (BMI, WC, SBP, DBP, UA, FBG, TC, HDL-C, LDL-C, Cr, and Urea) are presented as the means ± standard deviation and were analyzed by analysis of variance (ANOVA). Variables with a skewed distribution (age, TG, and GGT) are presented as the median (interquartile range) and were compared by the Kruskal-Wallis test. Categorical data (age group, regions, ethnic group) are presented as percentages and were compared by the x2 test. The tests were performed to compare the variables with the relevant groups. Multivariate logistic regression analyses were performed to calculate the odds ratios (ORs) for CRFs according to the GGT quartile, adjusting for age, UA, regions, and all other risk factors. It is worth mentioning that we used the JNC7 of hypertension in analyzing the association between serum GGT and CRF clustering. The P-values quoted are two-sided, and those values with P < 0.05 are regarded as statistically significant. All statistical analyses were performed with SPSS 16.0 software (SPSS Inc, Chicago, IL).

## Results

### General Characteristics of the Study Population

The baseline characteristics of individuals are shown (Table 1) according to sex-specific quartiles of GGT: the 25th, 50th, and 75th quartiles were 17 IU/L, 25 IU/L, and 43 IU/L for men, and 11 IU/L, 15 IU/L, and 23 IU/L for women. Men had a higher serum level of GGT than women did in each quartile. Regardless of gender, participants in the higher quartile of GGT were more likely to have significantly higher values for BMI, WC, SBP, DBP, UA, FBG, TG, TC, LDL-C, creatinine (Cr), urea, but not for HDL-C levels and eGFR (all P < 0.001). Except for Cr, Urea, and eGFR, whose values fluctuated, the rest of the indictors showed linear rising or falling trends from low to high serum GGT quartiles.

**Table 1:** Baseline characteristics of individuals according to sex-specific quartiles of GGT.

	Men				P value^a^	Women				P value^b^
	Q1(<17 U/L)	Q2(17–25 U/L)	Q3(25–43 U/L)	Q4(>43 U/L)		Q1(<11 U/L)	Q2(11–15 U/L)	Q3(15–23 U/L)	Q4(>23 U/L)	
n	2624	2617	2801	2673		2502	3253	3262	3165	
Age	33 (22–56)	42 (27–59)	46 (35–59)	44 (36–54)	<0.001	34 (25–44)	39 (27–52)	46 (34–58)	51 (42–60)	<0.001
BMI (kg/m^2^)	21.8 ± 2.73	23.1 ± 3.17	24.6 ± 3.28	26.0 ± 3.43	<0.001	21.7 ± 2.57	22.4 ± 2.97	23.6 ± 3.37	24.8 ± 3.62	<0.001
WC (cm)	75.8 ± 8.3	80.0 ± 9.4	84.5 ± 9.6	88.8 ± 9.5	<0.001	71.9 ± 7.5	74.2 ± 8.9	77.9 ± 9.4	81.8 ± 9.7	<0.001
SBP (mmHg)	122.8 ± 15.7	127.1 ± 16.9	130.0 ± 17.6	131.9 ± 17.1	<0.001	116.1 ± 15.5	120.0 ± 17.1	125.7 ± 19.5	130.8 ± 20.9	<0.001
DBP (mmHg)	75.4 ± 10.3	78.7 ± 10.8	81.6 ± 10.9	84.5 ± 11.4	<0.001	74.0 ± 10.0	76.1 ± 10.2	79.1 ± 10.8	81.8 ± 11.5	<0.001
UA (μmol/L)	322.7 ± 76.9	332.7 ± 77.5	350.1 ± 83.7	376.3 ± 86.8	<0.001	239.5 ± 57.1	245.9 ± 62.1	261.7 ± 64.5	283.2 ± 73.9	<0.001
FBG (mmol/L)	5.14 ± 0.78	5.27 ± 1.47	5.49 ± 1.31	5.73 ± 1.48	<0.001	5.04 ± 0.61	5.16 ± 0.79	5.35 ± 1.03	5.71 ± 1.49	<0.001
TG (mmol/L)	0.89 (0.65–1.21)	1.11 (0.80–1.57)	1.43 (1.01–2.12)	2.08 (1.37–3.17)	<0.001	0.84 (0.63–1.15)	0.98 (0.72–1.34)	1.21 (0.87–1.76)	1.55 (1.09–2.30)	<0.001
TC (mmol/L)	4.14 ± 0.91	4.47 ± 0.93	4.78 ± 0.96	5.17 ± 1.11	<0.001	4.27 ± 0.85	4.52 ± 0.93	4.84 ± 1.00	5.16 ± 1.06	<0.001
HDL-C (mmol/L)	1.33 ± 0.32	1.31 ± 0.33	1.27 ± 0.34	1.26 ± 0.35	<0.001	1.50 ± 0.33	1.48 ± 0.34	1.45 ± 0.34	1.42 ± 0.36	<0.001
LDL-C (mmol/L)	2.39 ± 0.78	2.62 ± 0.80	2.83 ± 0.83	2.93 ± 0.89	<0.001	2.37 ± 0.75	2.53 ± 0.81	2.78 ± 0.83	2.99 ± 0.90	<0.001
Cr (mg/dL)	80.7 ± 19.0	82.5 ± 15.2	83.5 ± 15.3	82.4 ± 15.8	<0.001	63.1 ± 14.9	62.9 ± 15.2	63.9 ± 13.1	65.0 ± 14.0	<0.001
Urea (mg/dL)	5.14 ± 1.49	5.27 ± 1.47	5.33 ± 1.47	5.24 ± 1.43	<0.001	4.54 ± 1.32	4.62 ± 1.42	4.83 ± 1.44	4.96 ± 1.43	<0.001
eGFR (mL/min/173 m^2^)	104.0 ± 21.4	98.3 ± 19.1	95.1 ± 17.4	97.1 ± 16.3	<0.001	105.7 ± 19.7	103.4 ± 20.2	98.2 ± 19.5	93.4 ± 19.9	<0.001
GGT (U/L)	13.2 (11.0–15.0)	20.1 (18.5–22.1)	31.4 (28.0–36.2)	66.4 (51.5–98.9)	<0.001	9.0 (8.0–10.0)	12.7 (11.8–13.6)	17.7 (16.0–20.0)	34.1 (27.0–52.0)	<0.001
Drinking	730 (27.8%)	926 (35.4%)	1225 (43.7%)	1575 (58.7%)	<0.001	104 (4.3%)	154 (4.7%)	159 (5.1%)	209 (6.6%)	<0.001
Dyslipidemia	795 (30.3%)	1192 (45.5%)	172 (61.5%)	2125 (79.5%)	<0.001	582 (23.3%)	1083 (33.3%)	1645 (50.4%)	2108 (66.6%)	<0.001
Hypertension^1^	402 (15.3%)	618 (23.6%)	868 (31.0%)	1016 (38.0%)	<0.001	255 (10.2%)	488 (15.0%)	809 (24.8%)	1082 (34.2%)	<0.001
Hypertension^2^	875 (33.3%)	1156 (44.2%)	1585 (56.6%)	1798 (67.3%)	<0.001	671 (26.8%)	1082 (33.3%)	1505 (46.1%)	1773 (56.0%)	<0.001
Diabetes	59 (2.2%)	90 (3.4%)	149 (5.3%)	223 (8.3%)	<0.001	9 (0.4%)	52 (1.6%)	111 (3.4%)	278 (8.8%)	<0.001
Current smoking	883 (33.7%)	1041 (39.8%)	1226 (45.2%)	1335 (49.9%)	<0.001	46 (1.8%)	71 (2.2%)	93 (2.9%)	130 (4.21%)	<0.001
Overweight	343 (13.1%)	718 (27.4%)	1268 (45.3%)	1614 (60.4%)	<0.001	261 (10.4%)	595 (18.3%)	1056 (32.4%)	1469 (46.4%)	<0.001

Data are shown as the mean ± standard deviation (SD), n (%); GGT, TG, and age were reported as medians (interquartile range). WC, waist circumference; SBP, systolic blood pressure; DBP, diastolic blood pressure; BMI, body mass index; UA, uric acid; TG, triglyceride; TC, total cholesterol; FBG, fasting blood glucose; HDL-C, high density lipoprotein cholesterol; LDLC, low density lipoprotein cholesterol; Cr, creatinine; GGT, gamma—glutamyltransferase; eGFR, estimated glomerular filtration rate. ^a^quartiles of comparison in men; ^b^quartiles of comparison in women.

^1^The prevalence of hypertension defined with JNC7 according to sex-specific quartiles of GGT.

^2^The prevalence of hypertension defined with 2017 ACC/AHA according to sex-specific quartiles of GGT.

Compared with women, men drank and smoked more on average. Whether smokers or drinkers, the proportions of women in each group were less than 10%. For men, however, the proportion could be more than half. Moreover, the percentages of each CRF in higher serum GGT levels were larger than those in the lower level. Among the CRFs, dyslipidemia accounted for the largest proportion for both genders in higher serum GGT (P < 0.001), followed by being overweight, having hypertension, currently smoking, and having diabetes (all p < 0.001).

It is obvious that the prevalence of hypertension increased as sex-specific quartiles of GGT for both genders according to the 2017 ACC/AHA, especially in high serum GGT quartiles.

### The Epidemiological Data of the Population

GGT levels were dichotomized by their upper quartiles (75th percentiles) to represent high (≥75th percentile) and low-to-medium (<75th percentile) levels of GGT. The demographic characteristics of the study individuals are shown in Table 2.

**Table 2:** Composition ratio of the sex-specific GGT <75th and GGT ≥75th among the study population for different groups.

	Men (n = 10715)		P value	Women (n = 12182)		P value
	GGT <75^th^	GGT ≥75^th^		GGT <75^th^	GGT ≥75^th^	
overall	8042 (75.1%)	2673 (24.9%)		9017 (74.0%)	3165 (26.0%)	
Age group (years)			<0.001*			<0.001*
18–34	3048 (83.6%)	600 (16.4%)		3422 (90.4%)	354 (9.6%)	
35–44	1320 (62.7%)	784 (37.3%)		2025 (74.9%)	677 (25.1%)	
45–54	1226 (64.1%)	686 (35.9%)		1526 (64%)	858 (36.0%)	
55–64	1262 (77.6%)	364 (22.4%)		1223 (61.6%)	764 (38.4%)	
≥65	1186 (83.2%)	239 (16.8%)		821 (62.1%)	502 (37.9%)	
Regions			<0.001^#^			<0.001^#^
South	4345 (78.6%)	1181 (21.4%)		4613 (76.0%)	1457 (24.0%)	
North	3697 (71.2%)	1492 (28.8%)		4404 (72.1%)	1708 (27.9%)	
Cities			<0.001^#^			<0.001^#^
Urban	3567 (79.5%)	921 (20.5%)		3823 (75.9%)	1216 (24.1%)	
Rural	4475 (71.9%)	1752 (28.1%)		5194 (72.7%)	1949 (27.3%)	
Ethnic group			<0.001*			<0.001*
Han	5319 (76.8%)	1610 (23.2%)		5738 (74.0%)	2014 (26.0%)	
Yi	631 (70.3%)	266 (29.7%)		815 (72.3%)	313 (27.7%)	
Hui	796 (80.9%)	188 (19.1%)		700 (76.4%)	216 (23.6%)	
Mongolian	286 (58.4%)	204 (41.6%)		496 (68.1%)	232 (31.9%)	
Korean	230 (65.0%)	124 (35.0%)		398 (74.0%)	140 (26.0%)	
Tibetan	226 (71.5%)	90 (26.0%)		349 (76.4%)	108 (23.6%)	
Tujia	268 (74.0%)	94 (26.0%)		237 (80.9%)	56 (19.1%)	
Miao	162 (73.0%)	60 (27.0%)		146 (76.8%)	44 (23.2%)	
Other	124 (77.0%)	37 (23.0%)		138 (76.7%)	42 (23.3%)	

*P-values were significant (<0.05) among groups.

^#^P-values were significant (<0.05) between groups.

For men, 8042 (75.1%) had GGT in the <75th percentile, and 2673 (24.9%) had GGT in the ≥75th percentile, whereas for women, the data were 9017 (74%) and 3165 (26%), respectively. The ratio of men and women in different levels of serum GGT differed by age group, region, and ethnic group (p < 0.001). The proportion of people with higher GGT levels was different in divided age groups; for men, the biggest percentage existed in the age range of 45–54 years, but for women, this occurred at the age range of 55–64 years. People in northern and rural areas tended to have upper quartiles of GGT, and for ethnic groups, Mongolians had the highest serum levels of GGT.

### Prevalence of CRF Clustering

The major CRFs that tended to cluster included diabetes, dyslipidemia, hypertension, current smoking, and being overweight. The age-stratified prevalence of clustered CRFs among participants in the GGT < 75th and GGT ≥75th levels are shown in Table 3. The prevalence of ≥1, ≥2, and ≥3 clustered CRFs was significantly higher in upper quartiles (75th percentiles) for men (95.4%, 79.4%, and 46.8%, respectively) and for women (82.5%, 52.4%, and 21.5%, respectively) than it was in low-to-medium (<75th percentile) levels of GGT in men (77.4%, 43.4%, and 16.7%, respectively) and women (51.4%, 21.2% and 5.8%, respectively). Furthermore, the prevalence levels of ≥1, ≥2 and ≥3 clustered CRFs in the upper quartiles group were higher than in the low-to-medium group for both genders and different age groups. In women, the prevalence of ≥1, ≥2 or ≥3 clustered CRFs increased with age. However, the prevalence of ≥1 clustered CRFs raised progressively with increasing age until 45 years old, and then decreased slightly, but for ≥2 or ≥3 risk factors, prevalence increased until 55 years old in men.

**Table 3:** Prevalence of clustered CRFs among participants in the GGT <75th and GGT ≥75th percentiles by gender and age. Data are presented as N (percent prevalence).

Clustered CRFs	Men		P-value^a^	Women		P-value^b^
	GGT <75^th^	GGT ≥75^th^		GGT <75^th^	GGT ≥75^th^	
**0 risk factors**						
overall	1815 (22.6%)	122 (4.6%)	<0.001	4379 (48.6%)	554 (17.5%)	<0.001
≥**1 risk factors**						
overall	6227 (77.4%)	2551 (95.4%)	<0.001	4638 (51.4%)	2611 (82.5%)	<0.001
**Age**, **y**						
18–34	1871 (61.4%)	562 (93.7%)	<0.001	836 (25.2%)	177 (52.8%)	<0.001
35–44	1111 (84.2%)	763 (97.3%)	<0.001	980 (48.4%)	504 (74.4%)	<0.001
45–54	1091 (89.0%)	659 (96.1%)	<0.001	1106 (72.5%)	747 (87.1%)	<0.001
55–64	1121 (88.8%)	348 (95.6%)	<0.001	1024 (83.7%)	712 (93.2%)	<0.001
≥65	1033 (87.1%)	219 (91.6%)	0.050	692 (84.3%)	471 (93.8%)	<0.001
≥**2 risk factors**						
overall	3492 (43.4%)	2122 (79.4%)	<0.001	1905 (21.1%)	1659 (52.4%)	<0.001
**Age**, **y**						
18–34	679 (22. 3%)	451 (75.2%)	<0.001	132 (4.0%)	70 (20.9%)	<0.001
35–44	662 (50.2%)	638 (81.4%)	<0.001	315 (15.6%)	261 (38.6%)	<0.001
45–54	713 (58.2%)	568 (82.8%)	<0.001	503 (33.0%)	455 (53.0%)	<0.001
55–64	756 (59.9%)	293 (80.5%)	<0.001	548 (44.8%)	518 (67.8%)	<0.001
≥65	682 (57.5%)	172 (72.0%)	<0.001	407 (49.6%)	355 (70.7%)	<0.001
≥**3 risk factors**						
overall	1344 (16.7%)	1252 (46.8%)	<0.001	526 (5.8%)	682 (21.5%)	0.001
**Age**, **y**						
18–34	176 (5.8%)	251 (41.8%)	<0.001	10 (0.3%)	19 (5.7%)	<0.001
35–44	236 (17.9%)	383 (48.9%)	<0.001	60 (3.0%)	91 (13.4%)	<0.001
45–54	302 (24.6%)	352 (51.3%)	<0.001	130 (8.5%)	163 (19.0%)	<0.001
55–64	335 (26.5%)	172 (47.3%)	<0.001	180 (14.7%)	227 (29.7%)	<0.001
≥65	295 (24.9%)	94 (39.3%)	<0.001	146 (17.8%)	182 (36.3%)	<0.001

^a^GGT <75th vs. GGT ≥75th in men; ^b^GGT <75th vs. GGT ≥75th in women.

### Multivariate Logistic Regression Analysis

Table 4 shows sex-specific associations of increasing GGT levels with CRFs and CRF clustering. Among men and women, the OR and 95% CI for CRFs and CRF clustering were calculated for each sex-specific GGT quartile, with the lowest quartile as the reference category, in multivariate logistic regression models after accounting for the effects of confounding factors. From low to high GGT quartiles, the incidence of each CRF increased except for smoking in women, which was only significant in upper quartiles.

**Table 4:** OR and 95% CI of sex-specific quartiles of GGT associated with major CRFs and clustered risk factors. Age, UA, alcohol, ethnicity, and all other risk factors were adjusted when estimating odds ratios (ORs) with 95% confidence intervals (CIs) of each variable.

	Men				Women			
	Q1(<17 U/L)	Q2(17–25 U/L)	Q3(25–43 U/L)	Q4(>43 U/L)	Q1(<11 U/L)	Q2(11–15 U/L)	Q3(15–23 U/L)	Q4(>23 U/L)
CRFs								
Dyslipidemia	1 (ref)	1.744 (1.549–1.963)*	3.061 (2.716–3.450)*	7.429 (6.486–8.510)*	1 (ref)	1.366 (1.201–1.554)*	2.212 (1.945–2.516)*	3.434 (3.008–3.922)*
Hypertension^1^	1 (ref)	1.341 (1.153–1.559)*	1.788 (1.546–2.067)*	2.611 (2.252–3.028)*	1 (ref)	1.206 (1.013–1.436)*	1.736 (1.468–2.053)*	2.254 (1.909–2.662)*
Hypertension^2^	1 (ref)	1.317 (1.170–1.483)*	1.959 (1.741–2.206)*	3.022 (2.665–3.428)*	1 (ref)	1.135 (1.007–1.280)*	1.607 (1.425–1.813)*	2.076 (1.833–2.350)*
Diabetes	1 (ref)	1.446 (1.023–2.045)*	2.200 (1.591–3.041)*	4.597 (3.347–6.316)*	1 (ref)	3.136 (1.527–6.442)*	5.483 (2.751–10.931)*	12.018 (6.108–23.644)*
Current smoking	1 (ref)	1.233 (1.093–1.391)*	1.355 (1.201–1.529)*	1.584 (1.397–1.796)*	1 (ref)	1.002 (0.680–1.474)^#^	1.161 (0.796–1.692)^#^	1.492 (1.032–2.157)*
Overweight	1 (ref)	2.105 (1.811–2.447)*	4.172 (3.609–4.823)*	7.417 (6.388–8.612)*	1 (ref)	1.519 (1.290–1.789)*	2.603 (2.223–3.047)*	3.853 (3.290–4.512)*
clustered risk factors								
1	1 (ref)	1.660 (1.454–1.895)*	3.258 (2.788–3.808)*	7.027 (5.683–8.689)*	1 (ref)	1.340 (1.185–1.515)*	2.407 (2.118–2.736)*	3.888 (3.377–4.476)*
2	1 (ref)	1.923 (1.693–2.185)*	3.707 (3.265–4.210)*	8.206 (7.128–9.447)*	1 (ref)	1.657 (1.386–1.982)*	3.071 (2.589–3.643)*	5.255 (4.435–6.226)*
≥3	1 (ref)	2.199 (1.804–2.681)*	4.107 (3.406–4.951)*	9.456 (7.848–11.392)*	1 (ref)	1.911 (1.321–2.764)*	3.853 (2.727–5.443)*	7.043 (5.019–9.881)*

^*^P < 0.05, ^#^P > 0.05.

^1^OR and 95% CI of sex-specific quartiles of GGT associated with hypertension defined with JNC7.

^2^OR and 95% CI of sex-specific quartiles of GGT associated with hypertension defined with 2017 ACC/AHA.

As the quartiles of GGT increased, the incidence of clustered CRFs also increased.

**Figure 2:**
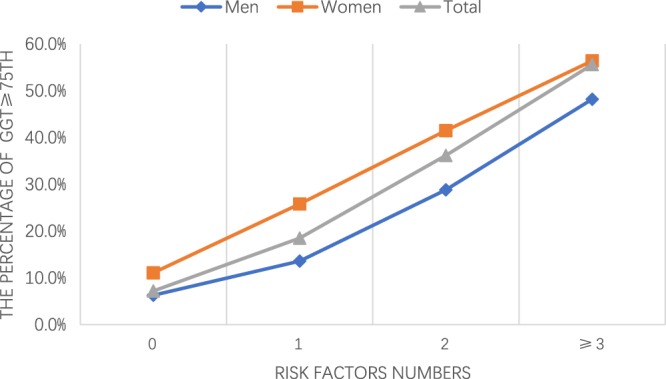
The percentage of GGT ≥75th for different numbers of risk factors.

The results of the multivariate logistic regression analysis for upper quartiles (75th percentiles) are shown in Table 5. Dyslipidemia, overweight, and diabetes were statistically significant CRFs and correlated with upper quartiles (75th percentiles) positively in both genders. Hypertension is also positively associated with upper quartiles (75th percentiles) especially with the new standard. In addition, the association between CVD risk factor clustering and different levels of GGT was further analyzed. In Fig. 2, it is clear that the proportion of GGT ≥75th was larger as the number of CRFs increased for both genders. However, the serum GGT of women tended to be influenced by CRFs more easily. As shown in Table 5, the results of the multivariate logistic analysis revealed that the CVD risk factor clustering was significantly and positively associated with upper quartiles (75th percentiles) when 0 CRFs were set as the reference category. We adjusted age, UA, drinking, ethnicity, and all other risk factors and found that participants with clustering of 1, 2, or ≥3 CRFs tend to belong to the upper quartiles (75th percentiles) than those having no risk factors in both genders. CRF clustering was associated with the higher ORs of upper quartiles in men than in women. Furthermore, there was a dose-response association between the number of CRFs and serum GGT. A stronger association of upper quartiles with ≥3 CVD risk factors was found in men (OR = 10.843, 8.748–13.441).

**Table 5:** OR and 95% CI of GGT ≥75th associated with major CRFs and clustered risk factors. Age, UA, alcohol, ethnicity, and all other risk factors were adjusted when estimating odds ratios (ORs) with 95% confidence intervals (CIs) of each variable.

CRFs	Men		Women	
	Unadjusted OR (95% CI) GGT ≥75th	Adjusted OR (95% CI) GGT ≥75th	Unadjusted OR (95% CI) GGT ≥75th	Adjusted OR (95% CI) GGT ≥75th
Dyslipidemia	4.530 (4.084–5.025)*	3.895 (3.487–4.352)*	3.439 (3.157–3.745)*	2.187 (1.985–2.408)*
Hypertension^1^	1.999 (1.820–2.195)*	1.709 (1.536–1.901)*	2.498 (2.280–2.738)*	1.483 (1.334–1.649)*
Hypertension^2^	2.515 (2.294–2.757)*	2.122 (1.920–2.344)*	2.251 (2.074–2.445)*	1.681 (1.537–1.839)*
Diabetes	2.365 (1.977–2.830)*	2.573 (2.1100–3.138)*	4.952 (4.076–6.016)*	2.904 (2.349–3,591)*
Current smoking	1.573 (1.439–1.720)*	1.325 (1.201–1.462)*	1.815 (1.452–2.267)*	1.365 (1.069–1.745)*
Overweight	3.739 (3.412–4.096)*	3.165 (2.859–3.504)*	3.219 (2.953–3.508)*	2.086 (1.896–2.295)*
clustered risk factors				
0	1 (ref)	1 (ref)	1 (ref)	1 (ref)
1	2.334 (1.892–2.878)*	2.096 (1.685–2.606)*	2.804 (2.498–3.148)*	2.087 (1.841–2.366)*
2	6.026 (4.936–7.356)*	4.853 (3.923–6.002)*	5.702 (5.052–6.437)*	3.412 (2.968–3.921)*
≥3	13.859 (11.360–16.907)*	10.843 (8.748–13.441)*	10.415 (9.013–12.036)*	5.344 (4.516–6.324)*

*P < 0.05.

^1^OR and 95% CI of GGT ≥75th associated with hypertension defined with JNC7.

^2^OR and 95% CI of GGT ≥75th associated with hypertension defined with 2017 ACC/AHA.

## Discussion

To our knowledge, our research is the first multicenter study to analyze the association between serum GGT and CRFs, as well as CRF clustering, in diverse Chinese provinces with a large and representative study population covering all adult age groups and including a plurality of ethnic minorities.

Our previous study revealed that serum GGT and uric acid levels were associated with impaired fasting glucose^16^. Kong, AP. *et al*. also reported that GGT and UA have a combined effect in association with obesity and other cardiovascular risk factors in the Chinese youth population^17^. In another previous study, we explored the relationship between hyperuricemia and clustering of CRFs in the Chinese adult population^23^. In the present study, we compared increasing GGT levels with CRFs and CRF clustering according to sex-specific quartiles of GGT, focusing on the relationship between the upper stratum (≥75th percentile) and lower stratum (<75th percentile) of GGT levels with CRFs in a population derived from a cross-sectional survey. The correlation between GGT and CRFs was further analyzed after adjusting several risk factors such as age, ethnicity, and drinking. UA was also included as a confounding factor and the results showed that GGT and CRF clustering was still significantly related after excluding the effect of these confounding factors.

In our study, people in the northern and rural areas tended to belong in the upper quartiles of GGT, which may be related to a different lifestyle, such as drinking more. In particular, Mongolians who live in the northwest of China had a higher prevalence of alcohol drinking^24^. Our data also indicated that Mongolians accounted for a bigger proportion in the upper serum levels of GGT compared with other ethnic groups. People who consume more alcohol tend to have higher serum GGT levels, a conclusion that is consistent with that of Tynjala J. *et al*.^25–28^. Men are more likely to drink than women. Similarly, current smoking slightly correlated with upper quartiles (75th percentiles) in both genders (all P < 0.05). Several reports support the notion of a detrimental interaction between cigarette smoking and alcohol consumption leading to elevated serum GGT levels, especially in men^29,30^.This may partly explain why these CRFs were more closely linked to men. One may reduce GGT levels by changing one’s lifestyle, such as by losing weight and abstaining from alcohol.

Our data suggests that the proportion of each risk factor increases as the quartiles of GGT increase. It was interesting to know dyslipidemia correlated with GGT levels mostly in men (OR = 3.895, 3.487–4.352, P < 0.05) and overweight men were more likely to have higher levels of GGT (OR = 3.165, 2.859–3.504, P < 0.05). Participants in the higher quartile of GGT were more likely to have significantly higher values of BMI and WC for both genders (P < 0.001). Similar to our study, a large cohort study confirmed that the risk of obesity (BMI ≥30 kg/m^2^) development was significantly and dose-dependently associated with serum GGT levels. This significant association was also found for WC-defined obesity (WC >90 cm)^31^; moreover, an increase in GGT concentrations is a sensitive and early biomarker of unfavorable body fat distribution^32^. Women with diabetes correlated with having higher levels of GGT mostly (OR = 2.904, 2.349–3.591, P < 0.05). The possible mechanism involves the GGT-mediated transfer of γ-glutamyl, an action that determines the levels of extracellular glutathione (GSH). GSH is a major antioxidant of cells; thus, GGT can antagonize the anti-oxidative effects of GSH, causing damage to vascular endothelial cells and leading to the hardening of arteries. Oxidative stress increases the level of GGT, resulting in reactive oxygen species that play a role in diabetes^33^. Furthermore, there are findings showing that serum GGT levels could be a predictor of the development of insulin resistance in Korean men^34^.

In this study, we found that there was a graded association between the GGT activity quartile and CVD risk factors. These CRFs were not independent and tended to cluster and associate with each other. We found greater CRF clustering associated with the higher odds ratio for higher serum GGT levels in both genders, especially for men. Many cohort studies have also shown that GGT may be a risk factor for total CVD mortality^35^ and that serum GGT activity may be an independent predictive marker of CVD, cardiac death, and cardiac transplantation^36,37^. Further studies are needed to confirm our findings and elucidate the exact mechanisms between GGT levels and CVD. GGT detection is associated with a number of advantages, including simplicity and low cost; it is likely to garner more attention in the future. In the previous literature, researchers used JNC7 to evaluate the relationship between hypertension and higher serum GGT levels. Here, we used both JNC7 and 2017 ACC/AHA in this article to evaluate the correlation between hypertension and high GGT levels, hoping to provide new ideas for controlling CVD by reducing GGT. However, there is no definitive way to reduce GGT, and there is no evidence that reducing GGT can reduce the incidence of cardiovascular events. A study reported that higher Q10H2 levels improved oxidative stress by reducing serum GGT activity in humans^38^. Thus, investigating the mechanistic role of GGT in cardiac diseases will be helpful in developing preventive strategies and treatment methods.

A major strength of the present study is that it is a population-based study with a representative sample of the general Chinese adult population which included a plurality of ethnic minorities; sufficient power was ensured by the large sample size in estimating serum GGT levels and the association with CRFs. In addition, we applied the standardized testing process in this multi-center study and all measurements in the study followed the same internal quality control program, which ensured the reliability and comparability of test results.

However, this study also has several limitations. First, this is a cross-sectional study, and thus cannot determine causality or the temporal relationship between serum GGT levels and CRFs. Second, the major CRFs such as diabetes, dyslipidemia, and hypertension were assessed mainly based on a single measurement of the corresponding parameters, although a detailed questionnaire survey including a history of self-reported disease was made. In addition, it is hard to avoid the reporting bias because many of the factors were self-reported, for example smoking and drinking. However, the relationship between serum GGT levels and CRFs found in this study is clear, and these limitations will not affect our conclusion.

## Conclusions

Our data highlight the association between higher serum GGT levels and CRFs in Chinese adults. We found that people with higher serum GGT levels tend to have a greater chance of CRFs and that there was a dose-response association between the number of CVD risk factors and higher serum GGT levels, especially in men, suggesting that serum GGT may serve as a valuable clinical marker of cardiovascular disease in China. Further studies are needed to elucidate the temporal nature of causality between serum GGT levels and CRFs and to evaluate the effects of serum GGT-lowering therapies on CVD prevention and outcome.

## Data Availability

The datasets generated and/or analyzed during the current study are not publicly available, but data may be available from the corresponding author on reasonable request.
